# Quantification of Circadian Movement of Small-Leaved Lime (*Tilia cordata* Mill.) Saplings With Short Interval Terrestrial Laser Scanning

**DOI:** 10.3389/fpls.2020.00984

**Published:** 2020-06-30

**Authors:** Ladislav Bakay, Ľuboš Moravčík

**Affiliations:** ^1^Department of Planting Design and Maintenance, Slovak University of Agriculture, Nitra, Slovakia; ^2^Department of Garden and Landscape Architecture, Slovak University of Agriculture, Nitra, Slovakia

**Keywords:** terrestrial laser scanning, plant movement, chronobiology, circadian rhythm, time series, 3-dimensional modeling

## Abstract

The goal of the study was to quantify and identify patterns in circadian movements of small-leaved lime (*Tillia cordata*) saplings with the help of terrestrial laser scanning (TLS). The movements were monitored every 60 min 24 h a day and every 30 min in the hour of sunrise and sunset. In order to exclude wind effects the monitored saplings were indoors. The resulting point clouds were used in creating a time series of branch and foliage movements with high precision. The circadian vertical movement of saplings was evaluated through target points, which has a potential of capturing the point-wise movement more accurately. Our results clearly show that small saplings move their branches and leaves during 24 h in complex ways and that is difficult to identify general patterns. Since we worked with small saplings and our movement threshold was 5 mm, we detected random fluctuation–oscillation as the most common movement in monitored saplings. The results highlight the potential of TLS measurements in support of chronobiology and the possibilities to analyze circadian movements of saplings in controlled environment.

## Introduction

Circadian movements of leaves were already known to the ancient Greeks (Bretzl, 1903). In one of the last publication of Charles Darwin The Power of Movement in Plants the process of nyctinasty was described for the first time (Darwin and Darwin, 1880). Plant movement was mainly studied on organ level (Zlinszky and Barford, 2018). Nocturnal movement of tree canopy was only recently discovered by using terrestrial laser scanning (TLS) (Puttonen et al., 2016). TLS is a 3D mapping tool of objects with high detail. TLS produces dense point clouds, which allow the quantitative analysis of objects (Dassot et al., 2011). Nocturnal plant movement was later quantified among 23 plant species (Zlinszky et al., 2017). Experiments showed also that plants have an internal mechanism for measuring time, since circadian movements were also present in plants kept in dark. Circadian oscillation and its molecular mechanism is described and extensively studied in *Arabidopsis* (Barak et al., 2000). Homologous gene sequences similar as in *Arabidopsis* controlling the diurnal rhythm of flowering time have been identified also in *Populus tremula* and *Castanea sativa* (Solomon et al., 2010). The movement of branches is probably an adaptation to light exposure. Nocturnal movement patterns are opposite to those during daylight (Mullen et al., 2006). Plant water balance (Chapin et al., 2002) and photoperiodism (McClung, 2006; Nozue and Maloof, 2006; Sysoeva et al., 2010) are the main drivers responsible for nocturnal movements in plants. All circadian plant movements can be divided in four basic movement pattern categories, (1) sleep or true circadian movement (circadian, light or internal clock controlled), (2) drift or non-cyclic unidirectional movement (non-circadian, a trend within the window of observation), (3) oscillation (short term fluctuation, typically superimposed on other types of movements) and (4) noise (random fluctuation around a mean zero within the expected noise limits) (Zlinszky et al., 2017). Circadian movement of trees is a variable phenomenon, probably controlled by a complex combination of anatomical, physiological, and morphological factors (Zlinszky et al., 2017). Zlinszky and Barford (2018) suggest that canopy movements are affected by an unknown pumping mechanism in the xylem described by Török (2017). Török (2017) presented his theory of novel hydraulic system in the xylem tissue, which moves solutes in pulses instead of a constant flow. Zlinszky et al. (2017) as well as Puttonen et al. (2016) described synchronized canopy movements in *Betula*, but also unsynchronized movements where the upper part of the tree canopy showed opposite movement as the lower part of the canopy. This unsynchronized movement could be explained by different turgor pressure in the canopy, which travels in pulses. Circadian crown movement differs in trees across different taxonomic groups. While gymnosperms show slight or no sleep movements, species as f.e. *Aesculus hippocastanum* or *Acer palmatum* have visible sleep patterns and have a circadian movement in an 8- to 12-h cycle (Zlinszky et al., 2017).

### Objective

Our aim is to gain an overview of circadian movement in lime tree saplings in containers focusing on vertical movement. Our method measures vertical movement of branch and leaves displacement in controlled environment with high precision TLS focusing on target points rather than on point cloud height percentiles. This is due to the fact that we dealt with smaller saplings and not mature trees.

## Materials and Methods

The study was carried out on July 4 to 5, 2017. The saplings were taken indoors 24 h prior the experiment for acclimatization in the new environment and were irrigated (full saturation of soil substrate).The first laser scanning was conducted at 12.00 pm and repeated every 60 min for 24 h. In the hour of sunrise (4:52 am) and sunset (8:51 pm) the laser scans were repeated every 30 min. The experiment was carried out in the facilities of the Faculty of horticulture and landscape engineering in Nitra (N48°18’49.540”, E18°4’44.420”) indoors in controlled environment. The measurement space had no artificial lighting. The lime tree saplings were grown in the plant nursery of the Faculty of horticulture and landscape engineering for 2 years, where they were watered on a daily basis. All saplings included in the study have thus been exposed to exactly the same weather and water conditions for the last 2 years and are a progeny of one mother tree. The study was conducted indoors to eliminate wind action. The experiment room did not get direct sunlight since the building has shading panels. Overnight we used shading with window blinds to eliminate streetlights in the room, where the laser scanning took place. We chose 4 yrs old lime tree (*Tilia cordata*) saplings grown in 3 l containers with TS 3 standard substratum (pH 5.5 to 6.0 + fertilizer 1 kg/m^3^) enriched by clay fraction (0–25 mm/m clay 20 kg/m^3^). The total number of the monitored saplings was 20. The average height of the saplings was 89 cm (height range, 58–109 cm). Each sapling had a reference code placed on the container (TK1 –TK20).

### Measurement Techniques

**Figure 1:**
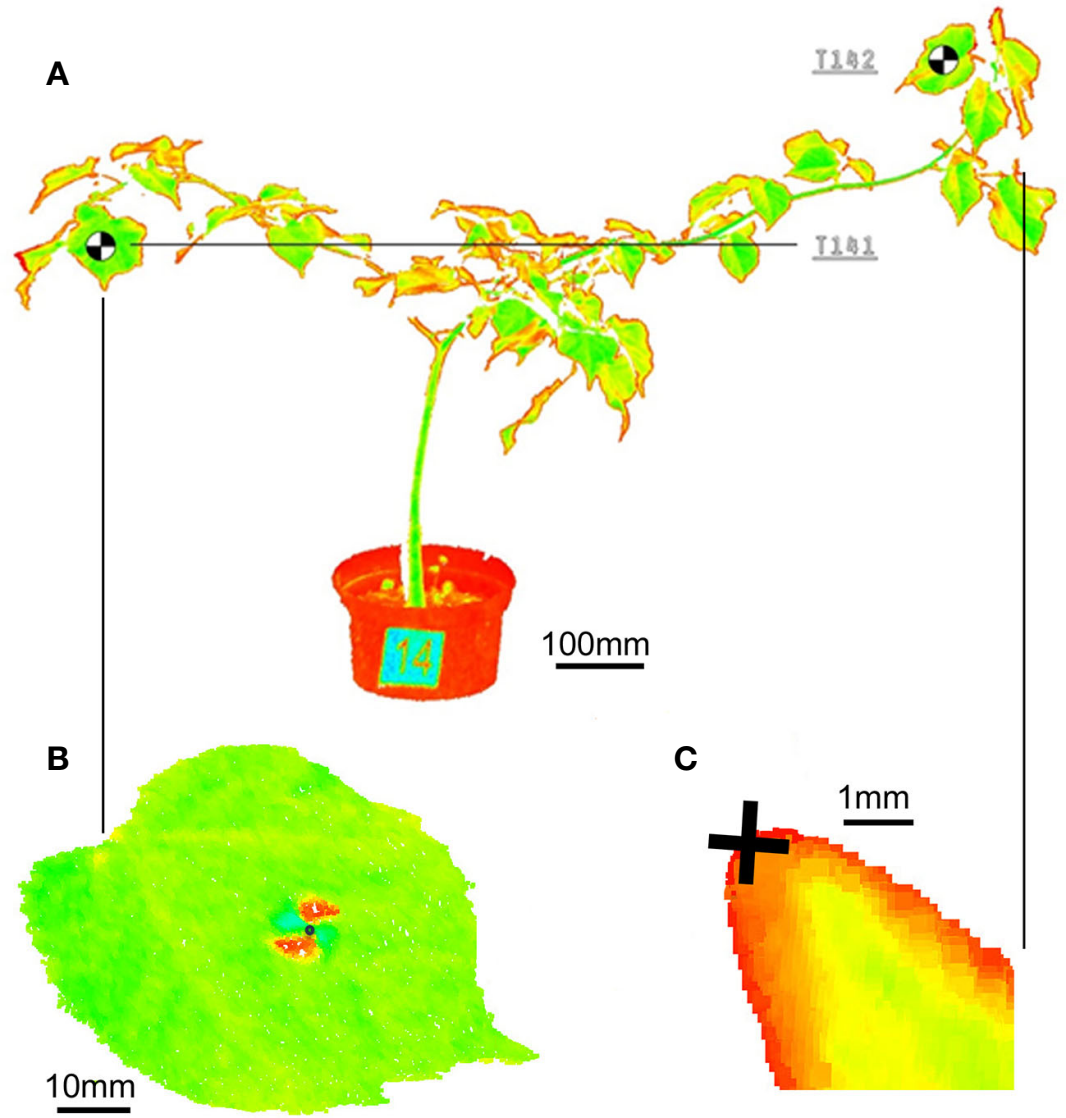
Point cloud of the **(A)** whole small-leaved lime sapling with visible number and target, **(B)** black-and-white target on the leaf and **(C)** 10x magnified view on a bud tip.

For the 3D point model of the plants under investigation a ultra-high speed pulse laser scanner Leica P20 enhanced by WaveForm Digitising (WFD) technology was used, operating a Class 1 infrared laser beam with 808 nm wavelength. Particular scans are performed with a restricted field of view - 360° (horizontal) and 45° to 135° (vertical). In order to decrease a size of point clouds the distance range to scanner was limited from 2.0 m (minimum) to 5.0 m (maximum). To achieve a sufficient density of the 3D point model for branches and leaves, the scanning resolution was setup to 1.6 mm at 10 m taken 13’33’’ of acquisition time for 1 scan. Relative spatial movements of leaves and branches were measured by the means of reference points placed on the defined positions of particular plants. For the investigation we have applied 30 targets (codes T11-T201) on leaves and selected 12 terminal buds on branches (codes TB2-TB20). The targets were printed on paper and glued to the leaves. The target weight did not influence the movement of leaves. We placed the targets on leaves which were the most distant from the main trunk. Reference points are represented by the black-and-white graphic marks in form of 10 x 10 mm rectangles with contrasted allocation of the target center. Reference points on the buds were the tips of the bud on selected branches (**Figure 1**). Data derived from terrestrial laser scanning represent the spatial XYZ coordinates and intensity of reflected light of the reference points observed on the leaves and buds measured in the constant 3D scanner coordinate system. Central points of targets were visually recognized on the output 3D model according to values of reflected light intensity. The accuracy of the XYZ coordinates determination is 1 mm. In first step the structured data was transferred from 3D scanner to PC workstation. Afterward, the HDS Cyclone 9.3.2 software was used performing a primary computer post processing for cleaning the raw point clouds from digital noises. Every point in the point cloud series is defined by XYZ coordinates and intensity of reflected light. We filtered the field of view of the scanner and point clouds were cleaned from noise manually in HDS Cyclone 9.3.2. Number of point cloud points in the scans fluctuated from 66976098 to 67754149. We did not use external reference markers on fixed targets since each scan was performed in identical coordinate system. The same application we used for the precise identification of the XYZ coordinates of reference points located on leaves and buds for all 26 scans. We focused on the vertical movement of the target centers and the bud tips. We evaluated only data from target points, which were visible on all measurements. Indoor air temperature and humidity were recorded with Humair 9 SN 123/14 relative humidity probe and PT100 temperature probe EasyLogGSM ultra low power data logger. These were set to collect data every 15 min. Light conditions were not measured. When taking consideration, plant movement we defined our noise threshold as 1 mm and movement threshold as 5 mm, because saplings have naturally a smaller absolute movement range as adult trees used in previous studies (Puttonen et al., 2016; Zlinszky et al., 2017). Mathematical and statistical data analysis was performed using the Statgraphics Centurion XVII software (StatPoint Technologies, USA, XVIII, license number: B480-E10A-00EA-P00S-60PO).

## Results

### Ambient Conditions

Wind speed and light conditions were not measured, since the experiment took place indoors without air conditioning. Air temperature decreased gradually from 27.3°C at 12:15 pm to 21.1°C at 4:30 am and increased next day to 26.9°C at 11:00 am. The windows of the room were oriented toward east and the room received indirect sunlight (dispersed light) from (sunrise) till 1:00 pm. Relative humidity oscillated during the 24 h between 42% and 47%.

### Observation in Tree Position Movement

**Figure 2:**
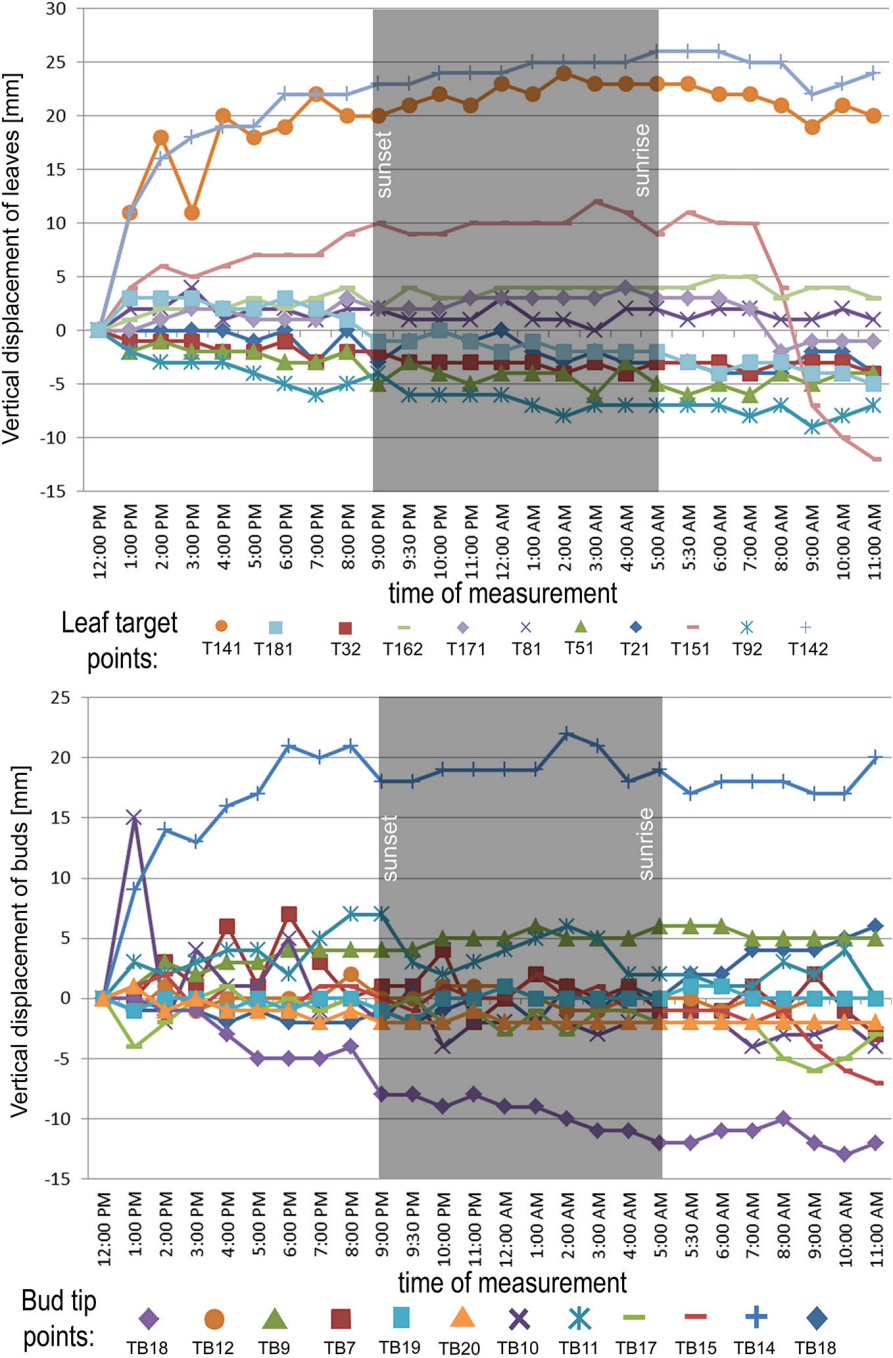
Vertical displacement of target points on leaves on monitored saplings during the time of measurement. Leaf target points with movement range below 5 mm in the measurement timeframe excluded from the figure. Vertical displacement of buds on all monitored saplings during the time of measurement.

**Figure 3:**
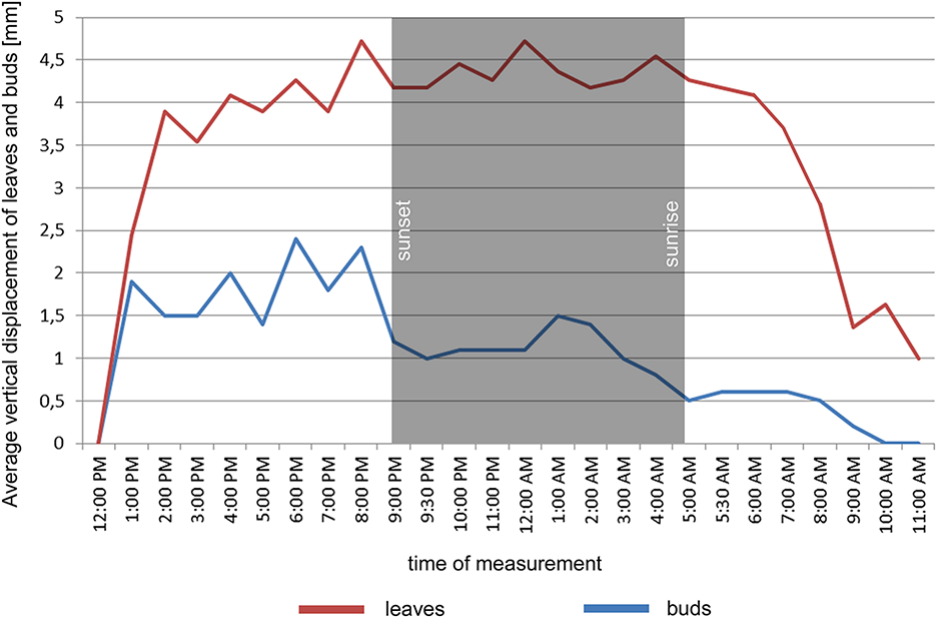
Average vertical displacement of buds and leaves on monitored saplings during the time of measurement.

**Figure 4:**
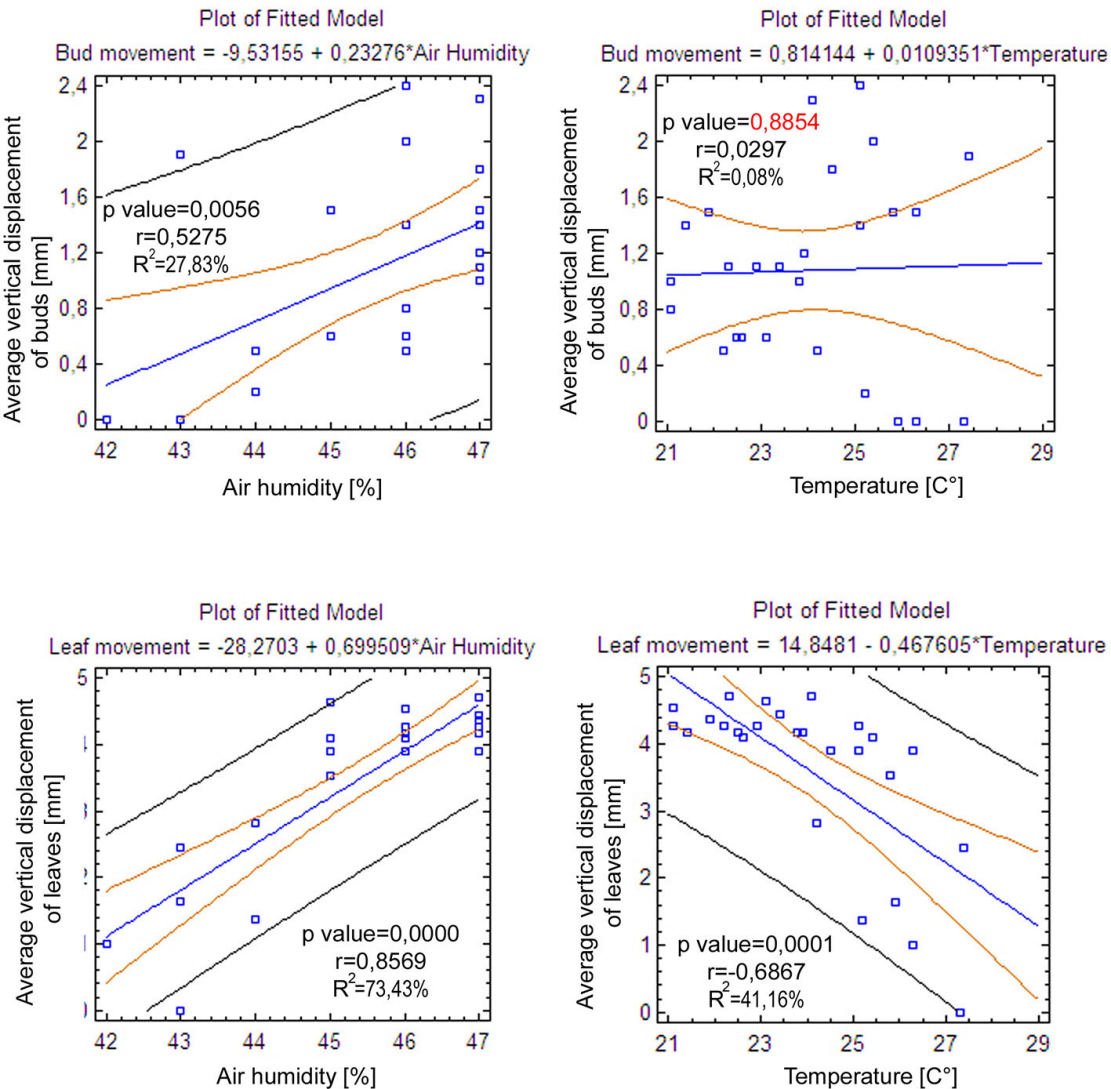
Linear regression models representing the correlations between average vertical bud and leaf movement with air humidity and air temperature.

In the point cloud series we identified 24 leaf target points and 12 bud tip points. Six leaf target points were not visible on all scans due to the orientation of leaves. The saplings showed various patterns in vertical movement measured on the terminal buds of the shoots (**Figure 2**). Bud tip point TB2 on sapling TK2 oscillated in a range of 4 mm and started a continual unidirectional upward movement after sunrise. This can be considered as a drift. TB2 was 6 mm above its starting position after 24 h. TB7 on sapling TK7 showed strong oscillation before sunset in a range of 8 mm with an upward trend. After sunset the oscillation was less intense (range 5 mm), but it had an opposite, downward direction. After 24 h TB7 was 3 mm below its starting position. TB9 on sapling TK9 shoved a continual unidirectional upward movement (drift) through the whole time series. TB9 moved after 24 h 5 mm upward from its starting position. TB10 on sapling TK10 oscillated intensively before sunset in a total range of 18 mm. After the sunset there was minor oscillation in a range of 2 mm. TB10 moved after 24 h 4 mm below its starting position. TB11 on sapling TK11 oscillated with and upward trend till sunset. After sunset TB11 lost 5mm in height. TB1 oscillated till the end of the measurements in a downward trend in a total range of 8 mm. TB11 returned after 24 h to its starting position. TB12 on sapling TK12 showed minor oscillation in range of 3 mm. TB12 returned after 24 h to its starting position. TB14 on sapling TK14 showed a strong upward, unidirectional drift till sunset. After sunset there was only weak oscillation till the end of measurements. After 24 h TB14 was 20 mm above its starting point. TB15 on sapling TK15 showed the opposite movement pattern as TB14. After weak oscillation in the range of 3 mm TB15 started after sunrise a downward drift and the height of TB15 decreased by 6 mm. After 24 h TB15 was 7 mm under its starting position. TB17 on sapling TK17 showed oscillation. Stronger oscillation with larger amplitudes appeared at 1:00 pm and at 8 am. After 24 h TB17 was 3 mm below its starting point. TB18 on TK18 showed unidirectional downward movement with minor oscillations approximately 1 h before sunset and after sunrise. After 24 h, TK18 was 12 mm below its starting point.TB19 on sapling TK19 and TB20 on sapling TK20 showed oscillation in the range of 3 mm. TB19 returned to the starting position within the measurement timeframe, but TB20 was 2 mm below its starting point at the end of the time series. The movement of the target points on the leaves was similar to the movements of the terminal buds but logically more intensive (**Figure 2**). Leaf target points T11, T12, and T13 on TK1 showed only minor oscillation in the range of 2 mm. T21 on TK2 showed oscillation before sunset in the range of 4 mm. The oscillation continued with a downward trend till 8:00 am T31 and T32 on sapling TK3 oscillated in the range of 4 mm but in opposite direction trends. T41 on sapling TK4 oscillated in the range of 2 mm. T51 on sapling TK5 showed oscillation with a downward trend in a range of 5 mm. T71 on sapling TK7 did not exceed the noise threshold. T81 on sapling TK8 showed oscillation in the range of 4 mm. T91 and T92 on sapling TK9 did not move synchronically. While T91 oscillated with an upward trend in the range of 2 mm, T92 showed downward movement interrupted with two minor movements upward, short before sunset and after 9:00 am. Neither T91 nor T92 returned to their starting positions. T111 and T112 on sapling TK11 and T121 on sapling TK12 showed minor oscillations in the range of 2 to 3 mm. T141 and T142 on sapling TK14 showed synchronous unidirectional upward drifts. After 24 h, T141 was 20 mm above its starting point and T142 was 24 mm above its starting point. T151 on sapling TK15 showed unidirectional upward drift, till sunrise, when it started to drift downward and ended below its starting point by 12 mm. T161 on TK16 showed minor oscillation in the range of 2 mm, but T162 on the same sapling oscillated with and upward trend within the range of 5 mm. After 7 am, the oscillation continued but with downward direction. T162 ended after 24 h 4 mm above its starting point. T171 on sapling TK17 had a similar movement pattern as point T162 but the downward movement after sunrise was more significant. T171 returned after 24 h 1 mm below its starting position. T181 on sapling TK18 showed oscillation with a downward trend. T181 was 5 mm below its starting point after 24 h. T191 on sapling TK19 showed oscillation in range of 5 mm, and its trajectory reminds a mild sinusoid.T201 on sapling TK20 oscillated only in the range of 2 mm. The average movement of buds and leaves showed a cycle but the movements were more intensive during the daylight (**Figure 3**). The average start and the end position of the measured points on the buds were similar after 24 h. The average movement of the buds showed a downward trend before the sunset (8:00 pm). The maximum displacement of the mean of all monitored points did not exceed 5 mm which is our movement threshold, although there is a downward movement after sunset, which could be identified as sleep movement. Despite the similar long-term growth conditions and the same genetical background, the movement of saplings was varied. We found a statistically significant positive correlation between average vertical leaf movement and humidity and also between average vertical bud movement and humidity. We also found a statistically significant negative correlation between average vertical movement of leaves and temperature (**Figure 4**). Our results suggest that the evapotranspirational requirements of the environment and the temperature determine the movement of lime saplings.

## Discussion

Our results clearly show that small lime saplings move their branches and leaves during 24 h in complex ways and that is difficult to identify general patterns. We identified movements as oscillations but also drifts. Sapling TK14 displayed in **Figure 1** was the ideal sapling for measuring vertical movements of branches, because of their length. Branch length is the most important factor, when selecting specimens in such experiments. Since we worked with small saplings and our movement threshold was 5 mm, we detected overall vertical movements close to the preset noise threshold. Our results are similar to the findings of Mullen et al. (2006), where canopy or branch movement occurs in both upward and downward directions during the night, and this movement is affected by light exposure. During the day, these movements are opposite. Our saplings showed stronger branch movements during the day and slight movement during the night. The average movement results show a clear cycle, since the measured target point average vertical movement returned to its starting position after 24 h, but the experiment should be repeated for a longer period than 24 h, with larger saplings of the same species, which have long branches to highlight these circadian movements. Our findings also suggest that sleep movement occurs in *Tillia cordata* Mill. in downward direction as revealed by Puttonen et al. (2016). Similar as observed for *Olea* and *Viburnum* in the study of Zlinszky et al. (2017). In our measurements, we identified movements defined as oscillations, which could support the theory of Török (2017). Since our experiment lasted 24 h, we can compare our results with other studies (12 h) only partially. We used also a different method of movement quantification of monitored saplings. We did not divide the point clouds of the canopy in height percentiles (Puttonen et al., 2016; Zlinszky et al., 2017) but we focused on target points to ensure high precision and point-wise accurate movement. For future research of circadian movement of trees we suggest to run trials indoors with larger saplings (around 2 m) and for a longer period (minimally 48 h). It is important that the trees have longer branches, where the movement is more visible. Also plants need a longer acclimatization period than 24 hours 213 for the new indoor environment. Lime tree saplings were suitable for monitoring circadian movements with TLS.

## Data Availability Statement

The raw data supporting the conclusions of this article will be made available by the authors, without undue reservation.

## Author Contributions

LB and LM performed the 3D scanning of the lime saplings. LM performed the post-processing and data evaluation. LB and LM designed the experiment and co-wrote the paper.

## Funding

This research was supported by AgroBioTech Research Centre built in accordance with the project Building “AgroBioTech” Research Centre ITMS 26220220180.

## Conflict of Interest

The authors declare that the research was conducted in the absence of any commercial or financial relationships that could be construed as a potential conflict of interest.
